# Key genes and molecular mechanisms related to Paclitaxel Resistance

**DOI:** 10.1186/s12935-024-03415-0

**Published:** 2024-07-13

**Authors:** Adel I. Alalawy

**Affiliations:** Department of Biochemistry, Faculty of Science, University of Tabuk, Tabuk, 71491 Saudi Arabia

**Keywords:** Paclitaxel, Resistance, ABCB1, TRAG-3/CSAG2 gene

## Abstract

Paclitaxel is commonly used to treat breast, ovarian, lung, esophageal, gastric, pancreatic cancer, and neck cancer cells. Cancer recurrence is observed in patients treated with paclitaxel due to paclitaxel resistance emergence. Resistant mechanisms are observed in cancer cells treated with paclitaxel, docetaxel, and cabazitaxel including changes in the target molecule β-tubulin of mitosis, molecular mechanisms that activate efflux drug out of the cells, and alterations in regulatory proteins of apoptosis. This review discusses new molecular mechanisms of taxane resistance, such as overexpression of genes like the multidrug resistance genes and *EDIL3*, *ABCB1*, *MRP1*, and *TRAG-3/CSAG2* genes. Moreover, significant lncRNAs are detected in paclitaxel resistance, such as lncRNA H19 and cross-resistance between taxanes. This review contributed to discovering new treatment strategies for taxane resistance and increasing the responsiveness of cancer cells toward chemotherapeutic drugs.

## Introduction

The mortality rate is increased worldwide due to cancer and the resistance to chemotherapeutic drugs. The number of deaths in 2020 was 10 million, and it is expected to increase to 16 million by 2040 globally. The uncontrolled mortality rate is a result of the chemotherapeutic resistance of cancer cells [1]. Chemotherapeutic resistance occurs as a result of cancer cells becoming tolerant to different chemotherapies [2]. Resistance could be categorized into primary and secondary: primary is caused by tumor cells before therapy exposure, while secondary is attributed to tumor adaptation to the treatment, for instance, elevated expression of target proteins [3]. Cancer drug resistance is commonly attributed to genomic alterations. Moreover, resistance machinery types include EMT, signaling pathway bypass, drug efflux activation, and drug entry impairment [4–7]. During chemotherapeutic resistance, tumor size is not affected by chemotherapy and the occurrence rate of relapse is increased after the initial positive treatment [8]. Breast, ovarian, gastric, and uterine cancers become chemo-resistant after being treated with chemotherapy for a long time, especially paclitaxel.

Paclitaxel, a naturally occurring compound, is used as a potent cytotoxic drug against different cancer types, including bladder cancer, breast cancer, non-small-cell lung cancer, esophageal, gastric, pancreatic cancers, and head and neck cancer [9, 10]. β-tubulin is a target of the paclitaxel action which causes microtubule stabilization, resulting in cell cycle arrest and apoptosis [11]. In addition, taxane induces cancer cell death by activating the cleavage of procaspases and proapoptotic markers. Initially, cancer cells are susceptible to paclitaxel treatment, but paclitaxel resistance develops over time through several mechanisms. Paclitaxel resistance is observed in cancer cells that may be intrinsic, whereas resistance was acquired in other cancer cells after an initial positive response [12]. Cancer cells may activate paclitaxel resistance mechanisms such as DNA mutations, thereby altering the metabolic mechanisms that induce drug resistance and degradation. Furthermore, resistance to paclitaxel in cancer cells occurs through the activation of efflux drug proteins, modification of molecular pathways involved in apoptosis, and upregulation of paclitaxel resistance-associated gene-3 (*TRAG-3/CSAG2*) expression [13]. It is essential to discuss the strategies of paclitaxel resistance, which refers to cancer recurrence in patients treated with paclitaxel. In this review, I identified how paclitaxel resistance is developed through drug efflux, cell death inhibition, genetic modifications, and modifying the biological conditions of the epithelial-mesenchymal transition (EMT) in cancer cells. This review discusses new taxane resistance mechanisms and cross-resistance mechanisms to give a chance to find treatment strategies to overcome resistance and improve anticancer efficacy.

## Different types of taxanes

### Paclitaxel structure related to its action

**Fig. 1:**
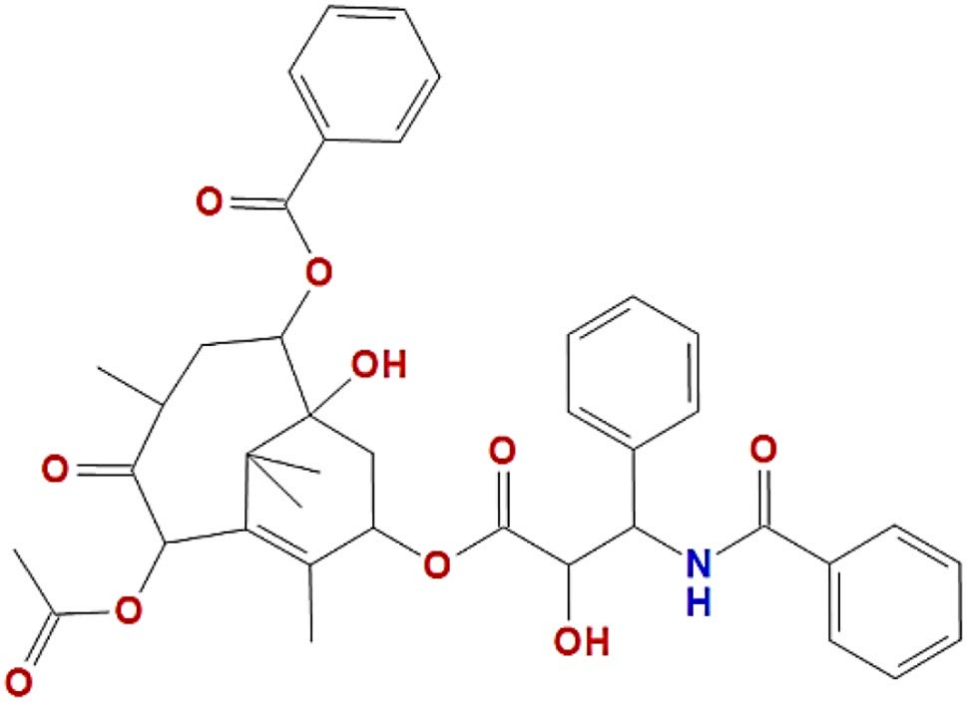
Chemical structure of paclitaxel

Paclitaxel is a natural product extracted as a white powder from the bark of a Pacific yew tree called *Taxus brevifolia*. Paclitaxel is a tetracyclic diterpenoid belonging to the class of taxane drugs that is characterized by its mode of action [14]. Taxane drugs, including paclitaxel, inhibit cancer cell proliferation by targeting microtubules. Paclitaxel is employed as an antineoplastic agent for the treatment of many malignancies [15]. The paclitaxel formula is C_47_H_51_NO_14_, as shown in Fig. 1, with a molecular weight of 853.9 g/mol. Paclitaxel possesses a taxane ring structure, whereby a homochiral ester is located at the C13 position, and a four-membered oxetane side ring is present at the C4 and C5 positions [16]. The C13 location in the paclitaxel structure exhibits a very active side chain, namely, a homochiral ester side chain. This side chain shows a strong affinity for microtubules, thus impeding their depolymerization process [16]. As a result, it limits the development of cancer cells by causing a stop in the cell cycle phases, specifically at the G2/M phase [5, 7, 17]. Guenard and coworkers (1993) observed that both a taxane ring and an ester sidechain in the paclitaxel structure have cytotoxic effects against cancer cells [18].

#### Mechanism of paclitaxel action

**Fig. 2:**
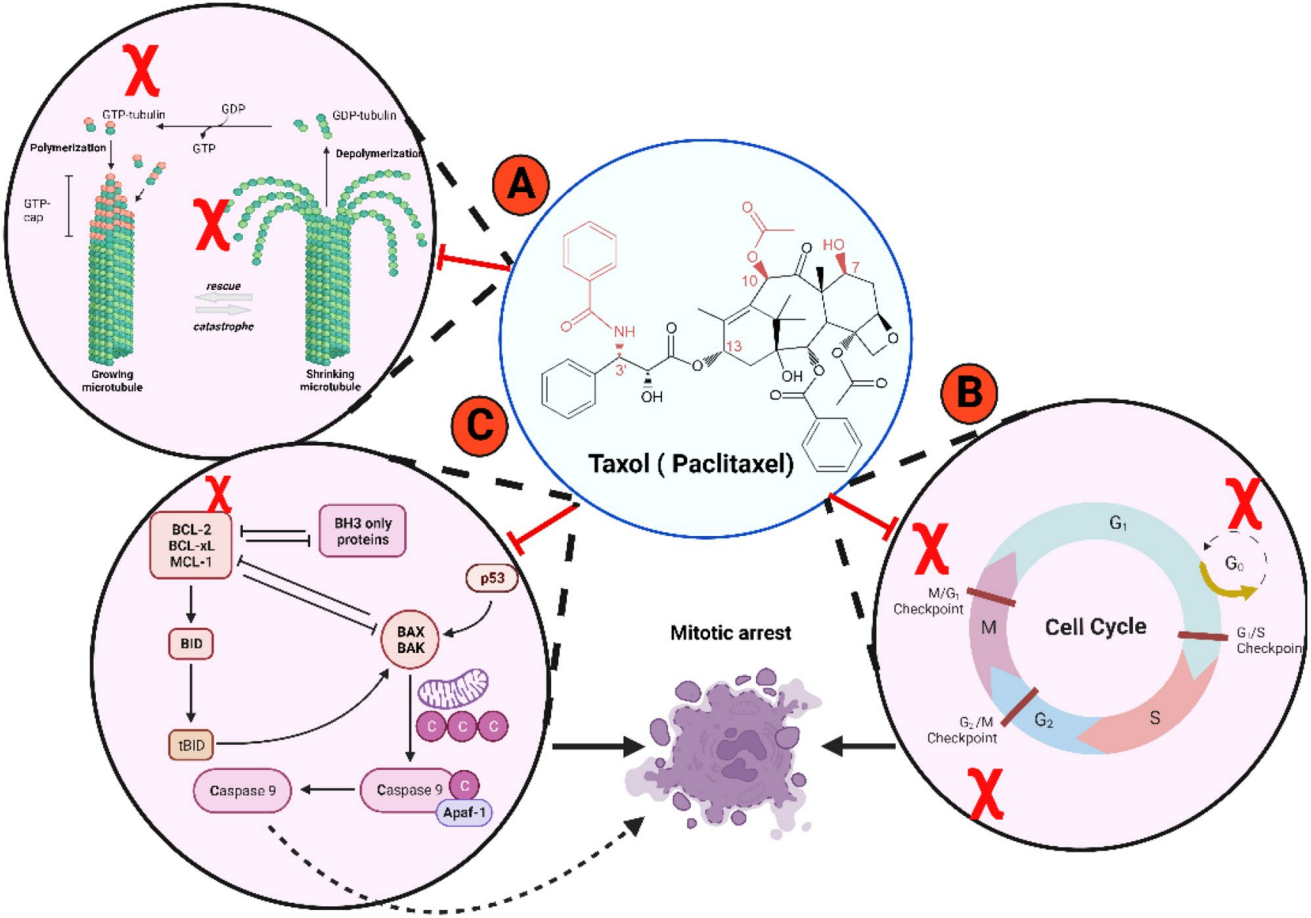
Mechanisms of paclitaxel mode of actions. **(A)** Paclitaxel targets 𝛽-tubulin heterodimers and stabilizes microtubule dynamics to prevent cell proliferation. **(B)** Paclitaxel causes microtubule depolymerization and arresting cells, especially in the G2/M phase and finally undergoing apoptosis. **(C)** Paclitaxel downregulates the expression level of bcl2 and drives the efflux of the mitochondrial cytochrome c resulting in caspase activation

Paclitaxel stabilizes the dynamics of microtubules, which contain cylindrical hollow structures with a diameter of about 25–30 nm. Microtubules are polymers of tubulin heterodimers, including α and β tubulins in dynamic balance [19, 20]. Microtubules are responsible for mitotic spindle formation throughout cell division and are needed for cell structure maintenance and motility of the cell with its intracellular cytoplasm. During the G2/M phase, the processes of tubulin biosynthesis and microtubule assembly take place continuously. There is a dynamic equilibrium between microtubules with their α and β subunits of tubulins arranged in a head-to-tail fashion. Under typical cellular equilibrium conditions, the net rate of tubulin assembly is equal to the net rate of tubulin breakdown, resulting in an unchanging microtubule length [21]. The microtubule contains a minus part that is anchored at the centrosome and ends that interact with cellular structures in the cytoplasm [22, 23]. Paclitaxel inhibits the assembly of microtubules and prevents cell division [24]. Additionally, it facilitates the formation of stable microtubules, specifically 𝛽-tubulin heterodimers, leading to the depolymerization of microtubules. This process prevents the progression of cells in the G2/M phase and eventually induces apoptosis, as shown in Fig. 2 [19, 25, 26]. N-terminal 31 amino acids of the β-tubulin subunit are the main target of paclitaxel interaction reversibly [25, 27]. Paclitaxel induces the formation of microtubules in vitro in the absence of GTP at 4 °C. Paclitaxel is observed to exert resistance mechanisms, including evading chemotherapeutic cytotoxicity that causes resistance and failure in chemotherapy [28].

Paclitaxel also activates various proapoptotic signaling pathways, such as the TLR-4 dependent pathway, c-Jun N-terminal kinase, P38 mitogen-activated protein (MAP) kinase, nuclear factor kappa B (NF-𝜅B), and Janus kinase- (JAK-) signal transducer and activator of transcription factor (STAT) pathway [29]. The activation of the P38 MAP kinase pathway by paclitaxel results in the phosphorylation of antiapoptotic markers, such as Bcl2, as well as the dephosphorylation of proapoptotic markers, including Bad and BAX [29]. Paclitaxel inhibits bcl2 (antiapoptotic marker) expression, which drives the expression of BAX (apoptotic marker) and mitochondrial efflux of cytochrome c, resulting in cleavage of procaspases into active caspases 3 and 9 and causes cell death, as shown in Fig. 2.

### Docetaxel

**Fig. 3:**
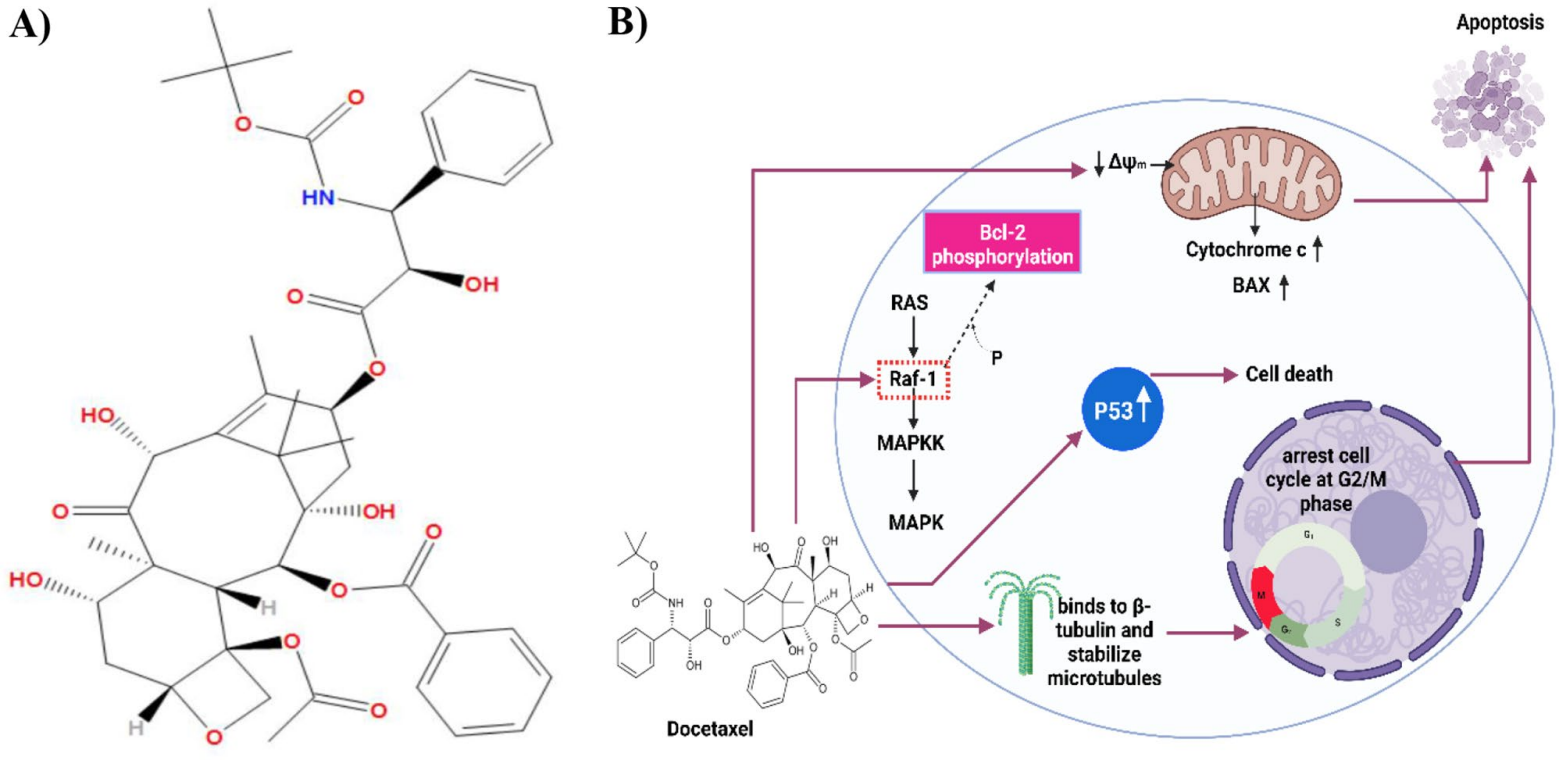
(**A**) Chemical structure of docetaxel. (**B**) Mode of action of docetaxel; docetaxel binds to β-tubulin causing the distribution of microtubules resulting arrest of cancer cells at the G2/M phase. Furthermore, it triggers apoptosis by causing disturbance in mitochondrial dynamics and drives the release of cytochrome c and upregulating BAX levels. Docetaxel Raf-1 kinase phosphorylates Bcl-2 resulting in apoptosis

Docetaxel (DTX) is a 2nd generation of the taxanes family is extracted from the needles of yew trees. Previous studies have shown that docetaxel and its derivatives have a significant anti-cancer activity more than paclitaxel by 1.3- to 12-fold [30] against a wide range of cancers including lung, ovarian, gastric, breast, prostate, and head and neck cancers [31–33]. The structure of docetaxel is different from the paclitaxel structure in the 3’-position of the lateral chain and the 10-position on the taxane ring as shown in Fig. 3A. Paclitaxel and docetaxel have the same binding sites and are different in the strength of suppressive activity on microtubule dynamics. Docetaxel has a greater binding affinity towards β-tubulin [34, 35]. In addition, the retention time of docetaxel on cancer cells is longer than paclitaxel. So, the effect of docetaxel is potent twice more than paclitaxel [34]. During mitosis, docetaxel binds to β-tubulin causing the distribution of molecular microtubule dynamics resulting in the inhibition of cytoskeleton functions and arrest cancer cells at the G2/M phase as shown in Fig. 3B [36]. Furthermore, docetaxel triggers apoptosis through phosphorylation of BCL2 and activation of caspase [37, 38]. Also, it inhibits cancer cell proliferation by decreasing the trafficking activity of the ERK1/2 pathway [39].

### Cabazitaxel

**Fig. 4:**
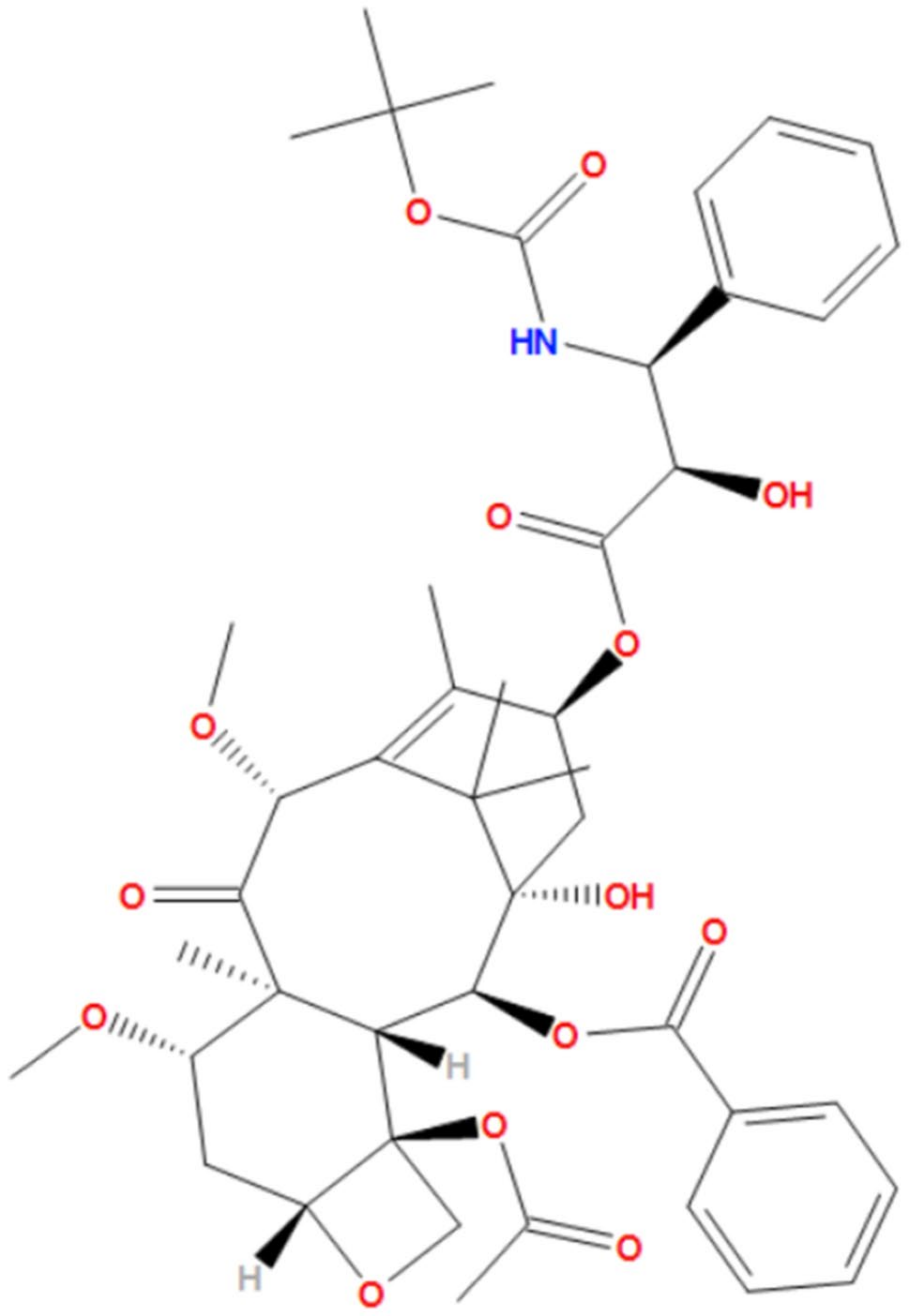
Chemical structure of cabazitaxel

Cabazitaxel (CBZ) is developed from docetaxel and its structure is different from docetaxel in its methyl groups at the 7- and 10-positions of the taxane ring as shown in Fig. 4 [40]. Cabazitaxel’s structure minimizes its affinity for drug efflux pumps including ATP-binding cassette and MDR1. Cabazitaxel is characterized by having a long intracellular retention time, which makes CBZ more potent than docetaxel in suppressing mitotic dynamics [41]. FDA approved cabazitaxel in 2010 as an anticancer drug. Cabazitaxel stabilizes microtubules by binding to the N-terminal amino acids of the β-tubulin subunit during mitosis. Therefore, cabazitaxel inhibits microtubule cell division and arrests the cancer cell cycle and its proliferation.

## Paclitaxel resistance with its key molecular mechanisms

Paclitaxel resistance is a complicated mechanism of multiple genes and multiple steps that develop cancer cells into aggressive cancer cells, causing a high mortality rate with advanced ovarian cancer, gastric cancer, and esophageal cancer [42–44]. The resistance of cancer cells can be attributed to their rapid and uncontrolled growth, leading to the development of several new blood vessels. These vessels enhance irregular blood flow and stimulate increased demand for blood supply, resulting in the development of hypoxic regions in patients with ovarian cancer. Chronic hypoxia leads to modifications in the tumor microenvironment of cancer cells adapted to keep cancer cell functions, including proliferation and progression, and subsequently decreases its chemosensitivity [45]. Chronic hypoxia induces genomic instability changes within cancer cells, and influences the expression level of the tumor suppressor gene (p53), vascular endothelial growth factor (VEGF), and angiogenin to inhibit programmed cell death and reduce cell cycle arrest, resulting in the emergence of cancer cell resistance towards different chemotherapies [46].

**Fig. 5:**
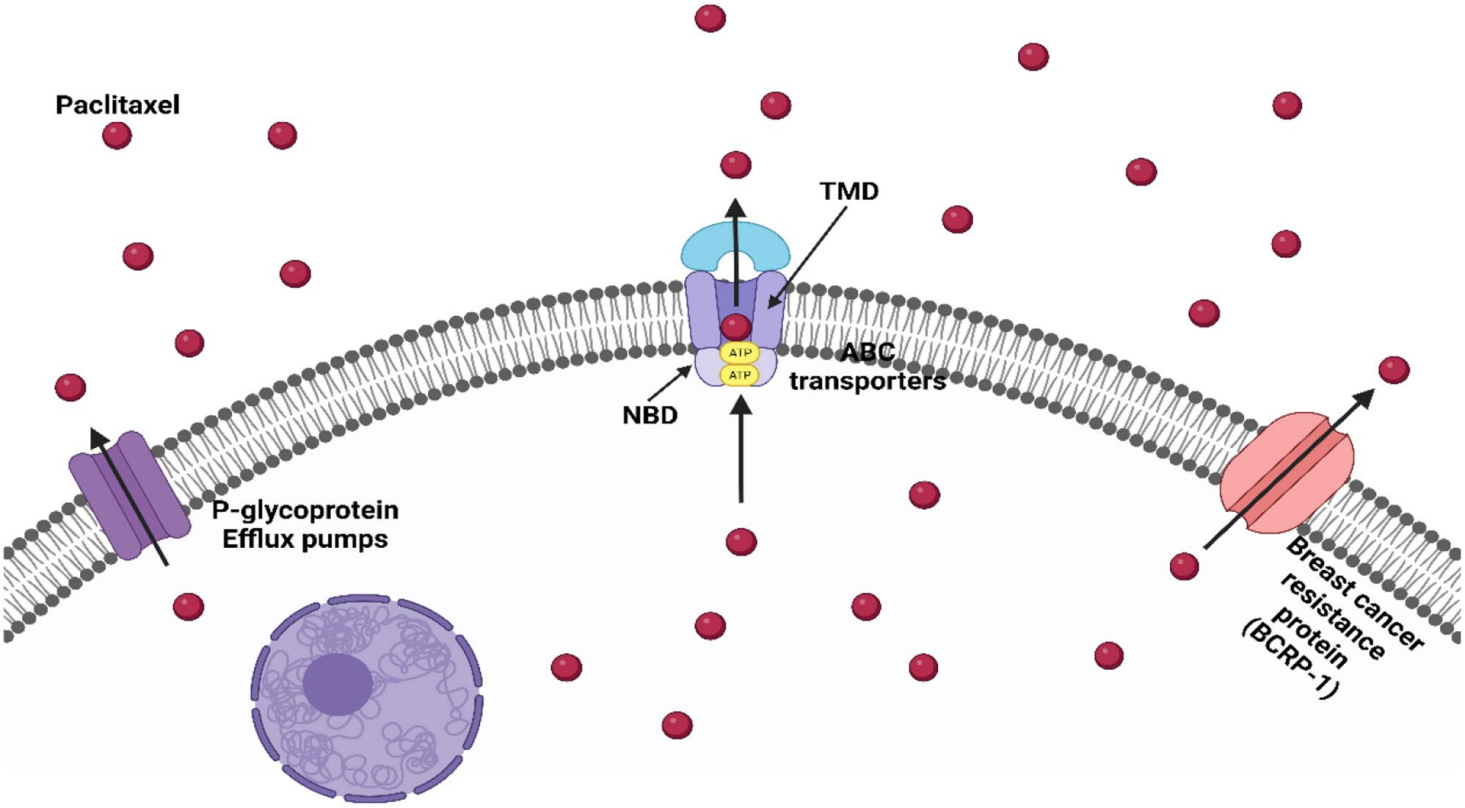
Resistant mechanisms of paclitaxel

In contrast, the development of resistance to paclitaxel is characterized by several modifications in mRNA and translation factors of protein, cellular oxidative stress, glycolysis, glutathione metabolism, and leukocyte transendothelial migrating pathways. Sherman-Baust and his coworkers (2011) observed that there is a total of 337 genes were extensively changed after treatment of resistant ovarian cancer cells with paclitaxel [47]. This alteration creates variations in drug effects, tumor microenvironment, mutations within microtubules, metabolism, and resistant tendencies toward chemotherapy among patients [47]. Moreover, several challenges reduce chemotherapeutic efficacy and enhance developing cancer cell resistance toward paclitaxel, including ABC transporter, P-glycoprotein, MDR-associated protein (MRP1), and breast cancer resistance protein (BCRP), as shown in Fig. 5. All these alterations inhibit cancer cell death, enhance the difficulty of treating metastasis, and avoid cancer cell targeting from chemotherapeutic drugs.

### Multidrug resistance (MDR)

Chemotherapeutic resistance or multidrug resistance (MDR) is related to the relapse of cancer cell responsiveness to many anticancer drugs and their mechanisms of action [48]. Paclitaxel develops cancer cell resistance through the upregulation of the *MDR* gene, which enables cancer cells to overcome chemotherapeutic drugs [49]. MDR exerts an external mechanism to modify the pharmacokinetics of chemotherapeutic drugs, modifying tumor microenvironment and causing changes within cancer cells. Ingrained or intrinsic MDR develops chemotherapeutic resistance by altering the cancer cell microenvironment. Acquired MDR occurs after exposure to chemotherapeutic drugs by modifying its pharmacokinetics or causing alterations within the cancer cells. Moreover, the *MDR1* gene translates ATP-binding cassette (ABC) transporters, including P-glycoprotein (P-gp), which functions as an efflux pump that enables cancer cells to deliver the needed substrates through cellular membranes [50]. Drug efflux is a crucial strategy that is responsible for paclitaxel chemoresistance. The other drug-resistant mechanisms include inhibition of drug penetration, modifications in drug targets, and alteration in the prodrug-activation mechanism.

### The ATP-binding cassette (ABC) transporters are implicated in the development of resistance to paclitaxel

The surface of resistant cancer cells is characterized by high expression of ABC transporter proteins. These transporters need energy to display their functions. ABC transporters are divided into three functional classes, including importers, exporters pump, and transporter-type proteins involved in DNA repair processes [51]. ABC importers are responsible for the uptake of biomolecules into the cell, whereas exporters are responsible for taking toxins and chemotherapeutic drugs out of the cancer cells. There are 7 ABC genes (ABCA–ABCG) that are divided and undergo their sequence homology and domain organization. ABC transporter proteins contain two domains, including the trans-membrane domain (TMD) and nucleotide-binding domain (NBD) [52]. The TMD in the ABC transporter identifies a large variability of substrates and causes conformational changes to drive passage biomolecules via the membranes in the presence of ATP. The structure and sequence of TMDs are variable, whereas the sequence structure of NBD is fixed and found in the cytoplasm where ATP-binding occurs. Ovarian and breast cancer overexpress ABCB1 and ABCC10 on their surface to pump paclitaxel out of the cancer cell and develop chemotherapeutic resistance for paclitaxel [53]. ABCB1 and ABCC10 are phosphor-glycoproteins encoded by MDR1 gene to decrease the therapeutic index of paclitaxel.

### Paclitaxel resistance associated with P-glycoprotein (PGP)

P-glycoprotein (PGP) is a multidrug efflux pump that consists of 14 targeting binding sites, including 12 TMDs and two ATP-binding sites. PGP is a multidrug transporter that is translated from the ABCB1 gene [54]. In normal healthy cells, PGP regulates the rate of the uptake of biomolecules, absorption, and efflux of foreign substances. Additionally, paclitaxel, daunorubicin, vincristine, doxorubicin, and vinblastine are transported through PGP by extracting the drug from the cytoplasmic side of the lipid bilayer directly [55]. The drug transfer through phospholipids from the inner to outer regions of the bilayer in PGP is facilitated by a unidirectional lipid flippase. The glycoprotein transporter known as PGP modulates the rate of drug penetration into cancer cells. There is an apparent relationship between the levels of PGP expression and the level of the emergence of chemotherapeutic resistance [55]. Breast cancer cells induce the PGP expression that actively pumps paclitaxel out of the cell in the presence of ATP against their concentration levels. This efflux induces breast cancer resistance and decreases its efficacy.

### Paclitaxel resistance associated with breast cancer resistance protein (BCRP)

The BCRP expression level is related to the presence of anticancer drugs, including paclitaxel, anthracyclines, camptothecins, and mitoxantrone, which induce the emergence of drug resistance [56]. BCRP consists of two binding domains, including the TMD and NBD [56]. The functioning homodimer of BCRP results from the bonding of two BCRP molecules with a disulfide bridge [57]. BCRP is expressed in normal cells of the gut, placenta, and bile canaliculi to eliminate toxins and xenobiotics from the cells [58]. BCRP enables normal cells to survive under hypoxia by keeping homeostasis levels of heme and folate [58]. Conversely, cancer cells upregulate the expression level of BCRP during hypoxic conditions and acquire chemotherapeutic resistance. Natarajan and coworkers (2012) observed that prostate cancer cell line 22RV1 is resistant to paclitaxel, methotrexate, and doxorubicin due to high BCRP and ABCG2 expression levels [58].

### Paclitaxel-resistance-associated gene-3 (TRAG-3/CSAG2) expression

Paclitaxel resistance in ovarian cancer is associated with the overexpression of gene 3 (*TRAG-3*) [59]. Previous studies demonstrated that normal cells have very low expression levels of this gene compared with ovarian cancer cells. The *TRAG-3* gene is located in the Xq28 region of the chromosome, specifically within a cluster associated with the MAGE (melanoma antigen) family of tumor antigens. This gene cluster comprises two coding exons [59]. TRAG-3 cDNA sequence contains an open reading frame of 333 bp that is translated as a protein product of 110 amino acids. *TRAG-3* gene does not drive paclitaxel-resistant phenotype directly, but it is a significant part of the multidrug-resistant mechanism of paclitaxel. Several types of cancer cells induce high expression levels of *TRAG-3*, including lung, breast, and melanoma cancer cells, which are related to paclitaxel resistance. Materna and his coworkers (2007) stated that paclitaxel treatment triggers the *TRAG-3* expression level in ovarian cancer [59]. Moreover, Duan and his coworkers (1999) reported that *TRAG-3* overexpression is observed in breast cancer cells resistant to paclitaxel, such as the MDA 435TR cell line, prostate, and myeloid carcinoma [60]. The relationship between the *TRAG-3* gene and the overexpression of the *MDR1* gene in cells with paclitaxel resistance was also investigated. After isolating the *TRAG-3* gene from resistant cells that overexpress the *MDR1* gene, it is observed that the *MDR1* gene expression is not related to paclitaxel resistance and that it does not drive paclitaxel toward resistance directly [61]. There is another gene called lipoprotein receptor-related protein (LRP), which is related to paclitaxel resistance and does not drive resistance [62]. The emergence of a drug-resistant phenotype needs coordination of the expressions of different individual genes to be sufficient to generate the phenotype. It is important to conduct several investigations to elucidate the *TRAG-3* role in tumorigenesis and drug resistance. In conclusion, it is considered a significant factor in the prognosis of paclitaxel resistance in ovarian carcinoma.

### Epithelial-mesenchymal transition-related EDIL3 gene and paclitaxel resistance

Cancer cells trigger metastasis, migration, and invasion by losing the protein adherent junctions between cancer cells to obtain a mesenchymal phenotype through the EMT process [63]. This process depends on integrin, TGF-β, and Wnt/β-catenin pathways. In addition, the Snail family contains SNAI1, SNAI2/Slug, and Twist members which could monitor the EMT process. The expression level of E-cadherin is inhibited by SNAI1, hence impacting the maintenance of the physical structure of cell junctions and the recruitment of cellular signaling pathways. Conversely, paclitaxel resistance is strongly related to the induction of EMT and the high expression levels of EMT-related genes, such as the *EDIL3* gene in breast cancer cells [64]. *EDIL3* gene encodes the extracellular matrix protein, which is a new regulator of EMT in tumor progression, adhesion, and migration [65, 66]. The expression of the *EDIL3* gene is associated with an increase in the expression of EGF-like repeats and discoidin I-like domains protein 3. The protein under consideration is an extracellular matrix (ECM) developmental protein with three EGF-like domains, including the RGD motif (Arg–Gly–Asp). This motif plays a crucial role in facilitating the protein’s interaction with integrins. Several research studies observed that the *EDIL3* expression level increased in different tumors characterized by poor prognosis, such as liver, bladder, pancreas, and bladder cancer [66]. The high expression level of *EDIL3* induces the expression levels of ERK and TGF-β after its interaction with the integrin αVβ3. Integrin αVβ3 induces brain metastasis in breast cancer patients. According to [67, 68], paclitaxel resistance is strongly associated with high expression levels of *EDIL3*. Knockdown of *EDIL3* by targeting the RGD motif induces the responsiveness of breast cancer and prostate cancer cells toward paclitaxel. In conclusion, *EDIL3* is a significant key regulator in paclitaxel resistance and EMT via autocrine or paracrine signaling in cancer cells.

### Cytochrome P450-related paclitaxel resistance

Metabolizing enzymes including Cytochrome P450 (CYP) enzymes contribute to paclitaxel metabolism such as CYP3A4 and CYP2C8 [69]. It is observed that invasive cancer cells have high levels of cytochrome P450 which induce cancer cell pathogenesis. There is a strong relationship between high expression levels of cytochrome p450 expression and the paclitaxel drug resistance. Increasing the level of paclitaxel metabolism limits the efficacy of paclitaxel by decreasing intracellular drug concentrations and developing drug resistance in cancer cells. Paclitaxel metabolism by cytochrome 450 increases its metabolites which have a low cytotoxic effect on cancer cells [70]. There are some challenges related to the activity of CYP enzymes to inhibit paclitaxel metabolism. Inhibition of the activity of CYP enzymes leads to inactivating the metabolism of other drugs which may cause critical drug–drug interactions. Cancer cells activate the cytochrome P450 and decrease paclitaxel cytotoxicity resulting in the development of paclitaxel resistance.

### Modulation of p53 related to paclitaxel resistance

Paclitaxel triggers cancer cell death via programmed cell death through a group of proto-oncogenes and tumor suppressor genes. Metzinger and co-workers (2006), observed ovarian cancer cell resistance with high levels of mutant p53 after the cells treated with paclitaxel or cisplatin. Interestingly, the modification in the steps of the regulation mechanism of apoptosis leads to the emergence of chemotherapeutic resistance. One of the tumor suppressor genes of apoptosis is the p53 gene which is included in some forms of apoptosis. Loss in the activity function of the p53 gene through missense mutation, deletion, and protein stabilizing enhances the development of chemotherapeutic resistance in cancer cells. The mutated p53 gene is called the TP53 gene which encodes mutated p53 protein. In addition, mutations in TP53 occur in all coding exons essentially including exons 4–9 which illustrate the DNA binding domain. This mutation not only diminishes p53 function but also enhances the acquisition of oncogenic characteristics. The new function of mutated p53 is promoting cancer cell metastasis, migration, and invasion. In addition, mutant TP53 enhances EMT and metastasis in cancer cells. Mutant p53 enhances cancer cell motility and invasion which require fibronectin-binding a5ß1 integrin to increase metastasis in H1299 cells. Also, mutant p53 increases the expression levels of integrin’s N-glycosylation patterns which enhances the interaction between cancer cells with the extracellular matrix. As a result of N-glycosylation, increasing the number of folding of membrane proteins in the endoplasmic reticulum as well as inducing metastasis.

### Modulation of metabolic enzyme expression related to paclitaxel resistance

Although paclitaxel has a significant role in the treatment of hormonal-resistant breast and prostate cancers, it loses its efficacy on cancer cells gradually and cancer cells become resistant to this chemotherapeutic drug [71]. Aoyama and co-workers (2017) declared that the cell membrane sphingolipids including sphingosine 1 phosphate (S1P), and ceramide have a significant role in the emergence of paclitaxel resistance [72]. Unbalance of the levels of ceramide and S1P induces the emergence of sphingolipid rheostat which leads to paclitaxel resistance. S1P is produced from sphingosine via sphingosine kinases (SPK1/2). Previous research studies observed that high expression levels of SPK1 and S1P trigger cancer cells tumorigenesis, survival, metastasis, migration, and invasion. In addition, overexpression of SPK1 induces chemotherapeutic resistance in leukemia and solid cancers. Not only the SPK1 enzyme but also the sphingomyelinase enzyme drives increasing the production of the levels of ceramide and apoptosis. Aoyama and co-workers (2017) observed that prostate cancer resistant to paclitaxel overexpress SPK1, and S1P as well as downregulating the expression level of ASMase [72]. Increasing the levels of SPK-1 enhances the expression levels of paclitaxel reflux out of the cell through ABC transporters and induces paclitaxel resistance. Knockdown of the expression level of SPK1 using siRNA decreases the levels of S1P in cancer cells resulting in inhibition of cancer cell proliferation and decreasing paclitaxel resistance [73]. Other inhibitors including PDMP and PPMP increase the level of ceramide in cancer cells resulting in cancer cell death and apoptosis in resistant prostate cancer [72]. In conclusion, decreasing metabolic enzyme SPK1/S1P levels can modulate the responsiveness of cancer cells towards paclitaxel.

## Long noncoding RNAs (lncRNAs) related to paclitaxel resistance

### lncRNA H19

Long noncoding RNAs (lncRNAs) regulate multiple resistance strategies. There are significant lncRNAs detected in paclitaxel resistance, such as lncRNA H19. High levels of lncRNA H19 drive the downregulation of the level of proapoptotic genes BIK to prevent cancer cell death and develop paclitaxel resistance [74]. High lncRNA H19 levels are observed in the triple-negative breast cancer cells resistant to paclitaxel. Han and coworkers (2018) observed that the downregulation of the lncRNA H19 expression levels increases the chemosensitivity of cancer cells and activates the Akt/phosphoAkt signaling pathway, resulting in cancer cell death [75].

### MA-linc1

There is a novel regulator of lncRNA called MA-linc1, decreasing the expression level of neighboring gene *Pur α*. The expression level of *Pur α* prevents cancer cell proliferation. Knockdown of the *Pur α* gene inhibits the activity of MA-linc1, resulting in inducing cancer cell sensitivity toward paclitaxel. Low level of survival rates of breast cancer patients is associated with a high level of MA-linc1 [76]. Bida and his coworkers (2015) observed that low MA-linc1 levels induce the sensitivity of cancer cells toward paclitaxel and overcome paclitaxel resistance [77]. Moreover, the lncRNAs HIF1A-AS2 and AK124454 induce proliferation, invasion, and paclitaxel resistance of MDA-MB-231 and TNBC cancer [78, 79].

### Linc-ROR

There is another new regulator of EMT called long intergenic noncoding RNA (Linc-ROR), which drives chemoresistance via miRNA interactions and TGF- β2 signaling [79–81]. The high expression level of Linc-ROR downregulates the expression level of E-cadherin and develops paclitaxel resistance in MDA-MB-231 cells [82].

### FTH1P3

There is another regulator of paclitaxel resistance called ferritin heavy chain 1 pseudogene 3 (FTH1P3). A high expression level of FTH1P3, which is resistant to paclitaxel, is observed in MCF-7 and MDA-MB-231 cells. The high expression level of FTH1P3 promotes the expression of drug transporter P-gp transporter, which drives the reflux of paclitaxel out of the cell and develops paclitaxel resistance. Furthermore, FTH1P3 targets miR-206, which is related to high levels of ABCB1 and develops paclitaxel resistance [83].

### NONHSAT141924

NONHSAT141924 is another long noncoding RNA that pertains to paclitaxel resistance. High expression levels of NONHSAT141924 have a significant role in upregulating the expression levels of Bcl-2, which leads to paclitaxel resistance [84].

## Key genes associated with taxanes resistance

**Fig. 6:**
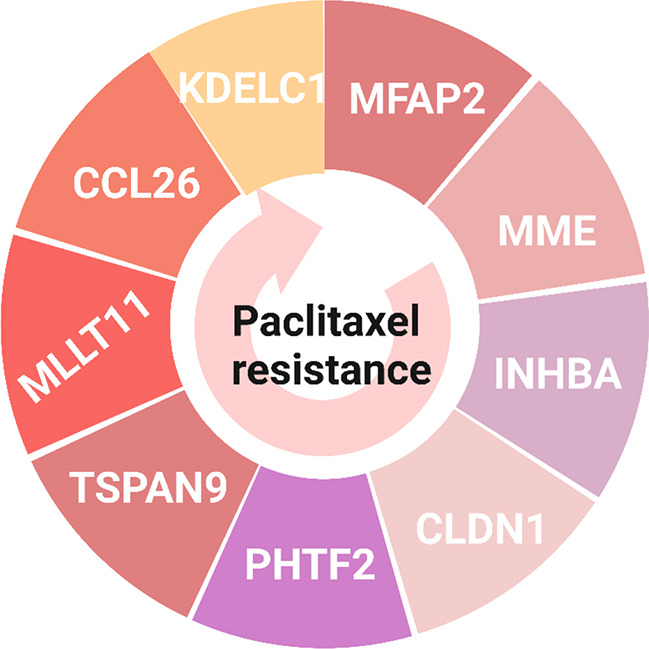
Key genes related to paclitaxel resistance

Treatment of cancer cells with paclitaxel for a long time leads to paclitaxel resistance. Shen and his coworkers (2021) identified the key genes related to paclitaxel resistance through LASSO analysis in esophageal squamous cell carcinoma (ESCC) [85]. There are nine specific key genes driving paclitaxel resistance in cancer cells, including microfibril-associated protein 2 (*MFAP2*), membrane metalloendopeptidase (*MME*), inhibin subunit beta A (*INHBA*), claudin 1 (*CLDN1*), putative homeodomain transcription factor 2 (*PHTF2*), tetraspanin 9 (*TSPAN9*), MLLT11 transcription factor 7 cofactor (*MLLT11*), chemokine (C-C motif) ligand 26 (*CCL26*), and glycosyltransferases (*KDELC1*) gene (Fig. 6) [85].

### *MFAP2, MFAP5* gene

*MFAP2* is one of the genes linked to paclitaxel resistance, which is located at 1p36.13 and consists of 10 exons [86]. *MFAP2* is a biomarker of ECM that enhances B16 melanoma cancer cell motility and invasion in vitro and in vivo research studies [87]. [88] used clinical samples to predict the association between *MFAP5* and the survival of ovarian cancer patients. The high expression level of *MFAP5* (microfibrillar-associated protein 5) induces the ovarian cancer resistance of paclitaxel and cisplatin [88]. [89] observed that *MFAP5* silencing in nude mice injected with luciferase labeled OVCA432 cells induces normalization of tumor vessels and improves the paclitaxel sensitivity.

### *MME* gene

Conversely, a low expression level of *MME* induces cancer cell resistance in prostate cancer [90]. MME is located at 3q21-27 and consists of 24 exons [91]. *MME* normally binds to phosphatase and tensin homolog deleted on chromosome 10 (*PTEN*), resulting in suppression of oncogenesis. Cancer cell resistance of paclitaxel is characterized by low levels of both *MME* and *PTEN*, driving vascular invasion and metastasis [90].

### *INHBA* gene

The *INHBA* gene is another gene observed in cancer cells resistant to paclitaxel [92]. *INHBA* gene is located at 2q35 and consists of two exons. Dysregulation of the *INHBA* gene induces metastasis and keeps mesenchymal phenotypes in cancer cells [93]. Chen and his coworkers (2019) observed that the *INHBA* gene induces the TGF-β signaling pathway, promoting cancer cell motility, invasion, migration, and consequently cancer cell resistance [94].

### *ITGA2* gene

Ma and his coworkers [95] observed that high expression levels of the *ITGA2* gene, which encodes integrin subunit α2, induce proliferation, metastasis, and the emergence of taxanes resistance in vitro. High expression of the *ITGA2* gene promotes the phosphorylation of forkhead box O1 (FoxO1) by facilitating the phosphorylation of AKT in taxane-resistant cells [95, 96].

### *CLDN1* gene

Moreover, the *CLDN1* gene is another paclitaxel-resistant gene observed in cancer cells at a high level. *CLDN1* gene is located at 3q28 and consists of four exons, which are expressed as membrane proteins responsible for tight junctions’ formation and monitor the transport of transendothelial, cell growth, and differentiation in normal cells [97]. Cancer cells contain dysregulated levels of *CLDN-1*, which trigger cancer cell invasion, aggressiveness, and resistance. Zhao and his co-workers [98] observed that high expression levels of *CLDN1* induce migration, invasion, and cisplatin resistance of NSCLC. High levels of *CLDN1* promote autophagy via increasing the expression levels of ULK1 phosphorylation and induce the emergence of cisplatin resistance in NSCLC [98]. *CLDN-1* is also involved in Wnt and Notch signaling, which has a significant role in the tumorigenesis, and resistance of cancer cells toward chemotherapeutic drugs [99].

### *PHTF2* gene

Furthermore, the *PHTF2* is a gene related to paclitaxel resistance in cancer cells, according to [85]. *PHTF2* is a gene located at 7q11.23 with 21 exons, which is observed to have a significant role in inducing tumorigenesis of gastric cancer cells by regulating fatty acid metabolism [100].

### *CCL26* gene

One of the genes related to paclitaxel resistance is *CCL26*, which is located at 7q11.23 with five exons [85]. In addition, the *CCL26* expression level is overexpressed in human osteosarcoma cells (MG63). *CCL26* enhances the invasiveness, proliferation, metastasis, and resistance in MG63 [101].

### *CCL2* gene

Natsagdorj and his coworkers [102] observed in vitro that cabazitaxel-resistant cell lines express high levels of *CCL2*, which induce invasion, migration, and chemoresistance in prostate cancer cells. *CCL2* induces not only the activator of transcription 3 (STAT3) but also AKT in prostate cell line. It is observed that there is a strong association between *CCL2* and *MDR1*. Activation of STAT3 upregulates the expression level of *MDR1* and induces the emergence of paclitaxel and cabazitaxel resistance in prostate cancer cells [102, 103].

### *TSPAN9* gene

*TSPAN9* is another specific gene related to paclitaxel resistance located at 12p13.33-p13.32 with 15 exons. The *TSPAN9* overexpression induces drug resistance in gastric cancer by downregulating the expression levels of PI3K proteins-related autophagy [104].

### *MAGE/GAGE* gene

Transfection of *MAGE/GAGE* gene into sensitive cancer cells drive the emergence of paclitaxel resistance in ovarian cancer cell line by 4-fold increase [105]. Acquisition of paclitaxel resistance related with increased expression of a variety of proteins and both neighboring and non-neighboring cancer antigens genes.

### *MLLT11* gene

*MLLT11* is also a specific gene-related paclitaxel resistance located at 1q21.3 with 2 exons. The *MLLT11* overexpression induces tumorigenesis, development, and resistance in endometrial cancer by targeting the Akt protein and consequently inhibiting the PI3K/AKT/mTOR signaling pathway [106].

### KDELC1 gene

*KDELC1* is one of the paclitaxel-resistant genes, which is located at 13q33.1 with 11 exons. Overexpression of *KDELC1* induces dysregulation of immune cell infiltration and driving Notch-related signaling [107]. High levels of *KDELC1* trigger cancer cell motility, invasion, progression, and consequently resistance [107]. Lueong and his coworkers [108] observed that after analysis of 10 patients who have a poor response to systemic chemotherapy that they have high expression levels of *KDELC1*. *KDELC1* activates many inflammatory pathways that are strongly associated with the pro-inflammatory microenvironment and result in the aggressiveness of PDAC tumors [108].Finally, it is important to study the genes related to taxane resistance to restore drug sensitivity and produce significant therapeutic strategies.

## Molecular mechanisms related to docetaxel resistance

### Alterations of microtubules

Docetaxel targets microtubule alterations including structural and functional [109]. Mutations of genes related to β- tubulin [110], high expression levels of βIII-tubulin [111], and alterations in the functional proteins associated with microtubules can modify the dynamics of microtubules and alter the capability of docetaxel binding [112]. Therefore, cancer cells become less sensitive to the toxicity of docetaxel and drive the emergence of docetaxel resistance.

### Upregulation of drug efflux transporter-related docetaxel resistance

 [113–115] observed that docetaxel upregulates the expression levels of ATP-binding cassette transporters including ABCC1, and ABCC4 in prostate cancer. ATP-binding cassette transporters efflux docetaxel out of the cancer cell and drive the emergence of docetaxel resistance. These transporters are characterized by drug specificity and included in chemoresistance causing drug failure to target cancer cells [109, 116]. Prostate cancer cells treated with docetaxel after a period express ABCB1, which is associated with TaxR genes automatically. ABCB1 knockdown induces the responsiveness of docetaxel-resistant cancer cells toward drug invitro [114]. Zhu and his co-workers [114] observed that the natural product apigenin inhibits the expression levels of ABCB1 and restores the sensitivity of docetaxel-resistant prostate cancer cells in vitro.

### Antioxidant activation

The toxicity of the taxane family is associated with the production of reactive oxygen species (ROS) inside cancer cells which leads to the activation of apoptosis [117, 118]. Taxane triggers variations in the mitochondrial membrane that induce the release of cytochrome C which activates ROS production and cleavage of procaspases into caspases and cancer cell death [119]. Then, cancer cells induce an antioxidant response that drives taxane resistance [117, 120]. High expression levels of antioxidant enzymes have been associated with docetaxel resistance. Furthermore, high expression levels of BIM-1 activate antioxidant response and minimize ROS production and cancer cell death in cells treated with docetaxel [117]. Therefore, it is observed that high levels of BIM-1 are associated with poor prognosis in prostate cancer and increased docetaxel resistance [117]. Interestingly, ROS production and antioxidant response activate pro-survival mechanisms and drive the emergence of docetaxel resistance. Also, a high expression level of superoxide dismutase 2 impairs the degradation of the arrestin-1-related insulin-like growth factor-I receptor (IGF-1R) which is associated with the reduction of ROS [121]. IGF-1 signaling has an obvious role in cancer proliferation and drug resistance [122].

### Cancer stem cells related to docetaxel resistance

Stemness properties of cancer cells enable cancer cells to be self-renewable and are related to drug resistance. The niche around cancer stem cells provides maintenance properties to cancer cells that enable them to resist chemotherapy [123, 124]. These maintenance properties include high survival capacity, high telomerase activity, upregulation of the expression levels of drug efflux transporters, EMT induction, and low levels of ROS production [124]. Lai and his co-workers [125] observed that resistant prostate cancer cells are characterized by the presence of CD44^+^ and/or CD133^+^ which make cells resistant to docetaxel toxicity. The high levels of CD44^+^ induce the HIPPO-YAP signaling pathway which induces metastasis, migration, and invasion and in turn chemotherapeutic resistance [125]. Furthermore, it is observed that prostate cancer stem cells which are resistant to docetaxel, are characterized by the presence of CD44^+^ and CD133^+^ and stemness-related genes [126]. The amount of ABCB1 transporters expressed in resistant cells depends on the activity of MDA-9, which is linked to the activity of the STAT3 and NOTCH/cMYC signaling pathways. The knockdown of STAT3 is a significant strategy to overcome docetaxel resistance in vitro [127, 128].

### SLCO genes and drug influx

Another promising taxane resistance mechanism is *SLCO* genes, which are responsible for reducing taxane concentration inside cancer cells and emergence resistance. It is observed that patients with castrate-resistant prostate cancer (CRPC) have low sensitivity towards taxanes associated with single nucleotide polymorphisms (SNPs) and a loss of activity in the solute carrier of organic anions (*SLCO*) genes [129–131]. *Slco1b2* Knockdown in mice is strongly associated with the high level of docetaxel in plasma which is not metabolized. SLCO proteins are important for transporting docetaxel into cells, and loss could lead to reduced intracellular docetaxel concentrations. [132] observed that impairment of docetaxel influx is related to the genetic variations of *SLCO* genes in vivo. From this point, there is no adoption to consider that the genetic polymorphism and expression levels are considered potential indicators for cellular taxane uptake or the emergence of resistance. Other studies observed that docetaxel resistance has emerged in prostate cell lines due to low expression levels of *SLCO1B3* [133]. The overexpression of *SLCO1B3* genes improves the sensitivity of prostate cell lines towards taxane [133]. The *SLCO* genes are related to the expression levels of efflux pumps on the cancer cell surface. These surface pumps drive the efflux of docetaxel out of the cell before its cytotoxic action [134]. de Morrée and his co-workers [135] observed that the influx and efflux pumps induce the emergence of docetaxel resistance in resistant patient-derived xenograft models of CRPC. It is important to focus on *SLCO* genes related to the influx pumps to overcome taxane resistance.

### Androgen receptor signaling

The androgen receptor is considered a ligand-dependent nuclear transcription factor that induces genes responsible for prostate cancer proliferation and differentiation [136, 137]. Androgen receptors are localized to the nucleus through metallothioneins [138]. Metallothioneins are proteins rich in cysteine which are responsible for mitotic spindle synthesis, metal homeostasis, and cellular protection that emerges from DNA damage and oxidative stress. In a normal cell, androgens signalling occurs after attachment to the androgen receptor, and metallothionein aid in its localization to the nucleus [139]. Taxanes stabilize metallothionein, resulting in the inhibition of androgen signalling. absence of androgen receptor localization is observed in the cells derived from docetaxel-treated CRPC patients. As a result, metallothionein is deduced to bind androgen receptors directly and the emergence of taxane resistance [140, 141]. It is observed that there is an inverse relationship between the amount of nuclear localization of the androgen receptor and the responsiveness of cells towards taxane. From this context, it is observed that the rate of sequestration of androgen receptors is related to the rate of the bundling of metallothionein which improves taxanes response [142]. Antonarakis and his co-workers [143] observed that there is a strong relationship between docetaxel resistance and androgen receptor signalling mechanisms. Once the androgen receptor is localized to the nucleus, Lysine-specific demethylase 5D (KDM5D) regulates the transcription of the androgen receptor. Knockdown of the KDM5D enzyme induces the signalling of androgen receptor and emergence of docetaxel resistance in cancer cells [144]. On the other hand, adding enzalutamide to taxane treatment decreases the effect of androgen receptor signalling on resistance. Enzalutamide as an anti-androgen inhibitor which block androgen receptor signalling. In conclusion, it is important to target androgen receptor signalling pathway as a new target to overcome taxane resistance.

### Epithelial-to-mesenchymal transition phenotype

The Epithelial-to-mesenchymal transition (EMT) phenotype has a crucial role in promoting cancer cell resistance and metastasis [145, 146]. EMT develops taxane resistance in castration-resistant prostate cancer CRPC cells. The low expression level of E-cadherin (an indicator of EMT) is observed in both untreated and docetaxel prostate cancer cells [147]. In addition, high expression levels of CD44 populations, vimentin, and zinc-finger e-box binding homeobox 1 (ZEB1) are observed in docetaxel-resistant CRPC cell lines [148]. [149, 150] observed that the knockdown of ZEB1 and decreased CD44 populations can induce the responsiveness of cancer cells towards docetaxel [150]. In addition, a high expression level of macrophage inhibitory cytokine 1 (MIC-1) is associated with EMT and docetaxel resistance [151]. MIC-1 is a member of the TGFβ family which targets decreased taxane resistance in vitro and in vivo [152]. Martin and his co-workers [153] observed that the treatment using the combination of both cabazitaxel and enzalutamide reversed EMT and decreased resistance. Targeting the EMT mediators is a promising strategy to overcome taxan resistance.

### PI3K/Akt Pathway

PI3K/Akt pathway is also another mechanism that induces survival in cancer cells by increasing the expression levels of activating kinases or downregulating the expression levels of the inhibitory regulators. Recent studies have observed that taxane resistance in prostate cancer is associated with irregular expression levels of PI3K/Akt [154–156]. [157, 158] observed that high expression levels of AKT and inhibition of phosphatase and tensin homologue deleted on chromosome 10 (PTEN) in PI3K induce chemoresistance of docetaxel in castration-resistant prostate cancer CRPC. So, it is observed that using the dietary flavonoid quercetin improves the sensitivity of resistant cells toward docetaxel by inducing apoptosis by decreasing the expression levels of phosphorylated Akt [159]. Furthermore, low PTEN expression levels are strongly associated with paclitaxel resistance. To overcome paclitaxel resistance and So, overexpression of PTEN induces the responsiveness of resistant cancer cells toward paclitaxel in vitro [158]. Docetaxel induces cancer resistance by increasing the activity of phosphorylated Akt depending on the dose of docetaxel. LY294002 is an inhibitor of PI3K/Akt signalling resulting in an improvement in the sensitivity of resistant cells to docetaxel and triggering apoptosis. Furthermore, NVP-BEZ235 targets the mammalian target of rapamycin (mTOR) results decreasing the expression levels of phosphorylated Akt, inducing cancer cell death invitro and inhibiting cancer cell proliferation in vivo [160]. As a result, NVP-BEZ235 is considered the most significant drug for inhibiting PI3K/Akt signaling and overcoming taxane resistance. In conclusion, targeting of PI3K/Akt pathway is the most significant mechanism to overcome taxane resistance.

### miRNAs-related taxane resistance

**Fig. 7:**
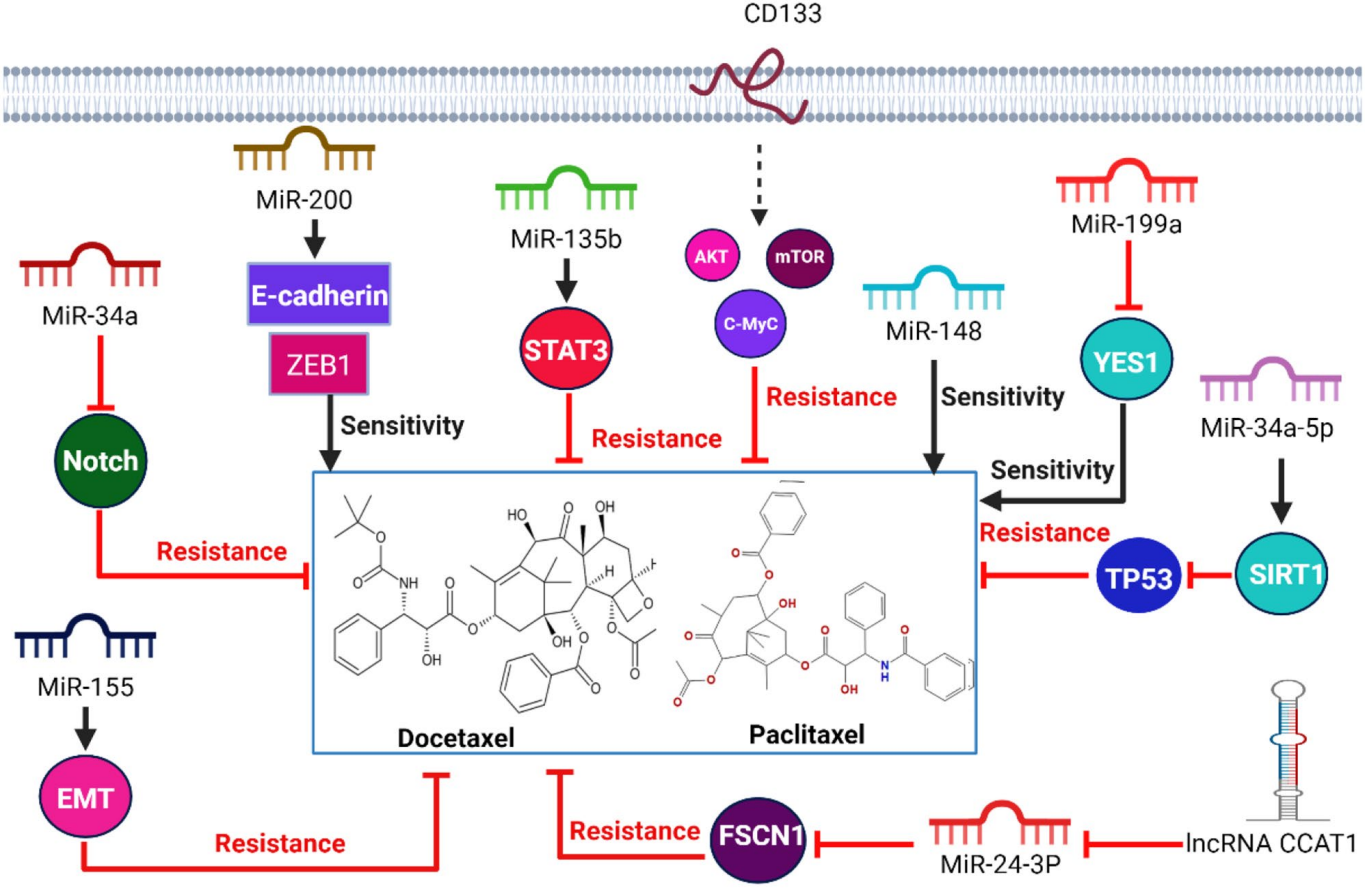
MiR-1273 g-3p, miR-34a, miR-135b, miR-34a-5p, miR-155, miR-24–3p, induces paclitaxel and docetaxel resistance, on the other hand, miR-148, and miR-200 restore the sensitivity of resistant cancer cells

MicroRNAs are considered endogenous non-coding RNAs with a short length of 19–24 nucleotides that regulate tumorigenesis [161, 162]. It binds to 3′-UTR of mRNA and reduces gene expression. In prostate cancer cells, miRNAs regulate cancer cell proliferation such as miR-1273 g-3p, miR-34a-5p, miR-148, and miR-34a as shown in Fig. 7. It is observed that overexpression of CD133 is displayed on the surface of the paclitaxel-resistant cells. CD133 induces the Akt/mTOR/c-Myc axis and the development of paclitaxel resistance [163]. MiR-1273 g-3p induces poor prognosis in prostate cancer cells by triggering cancer cell proliferation and metastasis [164]. It is observed that miR-34a has a low level in cancer cells resistant to paclitaxel. In normal cells, miR-34a targets the NOTCH signaling pathway that involves the BCL-2 family, but its downregulation induces paclitaxel resistance in cancer cells in vitro [165]. MiR-34a-5p induces cancer cell proliferation and triggers paclitaxel resistance in prostate cancer by inducing the expression levels of Human sirtuin1 (SIRT1) and suppressing TP53 expression [166]. In addition, overexpression of miR-135b induces paclitaxel resistance and aggressiveness of NSCLC by directly targeting the 3′-untranslated region (UTR) of the deubiquitinase CYLD, thereby modulating ubiquitination and activation of NF-κB signaling. Interleukin-6 (IL-6)/STAT3 could bind to the promoter of miR-135b results elevating its level of expression [167]. Also, high levels of miR-155 induce paclitaxel resistance in vitro. MiR-155 overexpression induces exosome secretion acquires epithelial-mesenchymal transition characteristics and promotes a high rate of migration and metastasis in breast cancer [168]. lncRNA CCAT1 has an oncogenic role by inhibiting the expression of miR-24–3p which minimizes the level of fascin actin-bundling protein 1 (FSCN1) that induces the paclitaxel resistance [169].

On the other hand, microRNAs could improve the sensitivity of cancer cells towards drugs. MiR-148 for example is used to improve the responsiveness of resistant cells towards paclitaxel [170, 171]. MiR-200 restores docetaxel sensitivity by increasing E-cadherin and ZEB1 expressions which induce apoptosis in prostate cancer [116, 147]. In addition, low expression level of miR-199a has a tumor suppressor role in prostate cancer. MiR-199a inhibits the expression level of YES1 (YES Proto-Oncogene 1) to overcome paclitaxel resistance [172]. In conclusion, microRNA expression levels are considered as chemotherapeutic targets to overcome paclitaxel resistance.

## Molecular mechanisms related to cabazitaxel resistance

Despite cabazitaxel’s potent cytotoxicity which is more than both paclitaxel and docetaxel, there is a substantial cross-resistance remaining. Taxanes, including cabazitaxel, activate efflux transporters such as ABCB1/P-gp [173–176]. It is observed that cabazitaxel is the most active taxane drug in expressing efflux transporters, including ABCB1, but not ABCC2, ABCC10, or ABCG2. Furthermore, any alterations in microtubule dynamics and composition have been observed in taxane drugs [166, 177, 178]. It was also observed that non-ABCB1 taxane resistance is associated with high expression levels of the β-tubulin isotype (TUBB4) in leukemia [179]. The cabazitaxel-resistant ovarian and breast cancer cells express high expression levels of the content of class III (TUBB3) in the presence of a P-gp inhibitor (PSC-833). Upregulation of TUBB3 enables MCF-7/ CTAX-P to acquire mesenchymal properties which are associated with cabazitaxel resistance [180–182]. It was also confirmed by [183–185] who observed that cabazitaxel resistance is a result of high expression levels of class III β-tubulin. [186–188] used siRNAs to inhibit TUBB3 expression to restore taxanes sensitivity. In addition, [189] observed that the knockdown of TUBB3 restores the sensitivity of the MCF-7/CTAX-P cell line towards cabazitaxel.

Other studies reported that knockdown of *BRCA1* expression is one of the significant causes of cabazitaxel resistance in breast, lung, and ovarian cancer cell lines [190–192]. Both *BRCA1* and *BRCA2* are genes that express proteins responsible for repairing damaged DNA. Furthermore, *BRCA1* is observed to control microtubule dynamics and binding of cabazitaxel to microtubules, impairing its effect as a stabilizer of microtubules and inducing the emergence of cabazitaxel resistance in A549 lung cancer [193]. Silencing of the *BRCA1* expression increases the cabazitaxel resistance by 4-fold in MCF-7 cells [194]. Targeting of EMT, level of *BRCA1*, and high level of class III β-tubulin are significant strategies to overcome cabazitaxel resistance.

## Cross-resistance between taxanes

Cross-resistance is considered as acquired resistance that is defined as a reaction to chemotherapy and results in resistance to other drugs. Cross-resistance may depend on the genetic profile of the patient [195]. The risk of cross-resistance emerges after sequential treatment of two chemotherapy have the same mode of action and results that the second drug might lose its efficacy [196]. The cross-resistance may be observed between paclitaxel and docetaxel or between docetaxel and cabazitaxel.

### Cross-resistance between paclitaxel and docetaxel

There are large cross-resistance is observed after using paclitaxel and docetaxel as a combination to treat metastatic gastric cancer [197]. Asić and his co-workers [3] observed among 484 patients with metastatic gastric cancer who received both paclitaxel and docetaxel as a combination therapy that the 2nd taxane achieved a response rate of 5% and an overall disease control rate of 17.9%. These results demonstrate a large degree of cross-resistance between paclitaxel and docetaxel [197]. So, after the failure of treatment by other types of taxane in gastric cancer, both paclitaxel and docetaxel should not be routinely administered for metastatic gastric cancer [197]. Also, Valero and his coworkers [198] evaluated the efficacy of docetaxel in patients who suffered from paclitaxel-resistant breast cancer (MBC). They observed that 7 patients from a total of 44 patients had partial responses and 1 patient responded completely with severe adverse effects. It is observed that docetaxel has a higher potency than paclitaxel in promoting phosphorylation of bcl-2, which results in high efficiency in inducing apoptotic cell death. Paclitaxel resistance mechanisms involving microtubule binding resistance may still be sensitive to docetaxel if the bcl-2 phosphorylation mechanism remains sensitive to docetaxel [198, 199]. If the bcl-2 phosphorylation is not sensitive to docetaxel this means microtubule binding resistance and the emergence of cross-resistance. Docetaxel induces the production of interferon-α and tumor necrosis factor-α (TNF-α) which downregulate cell death in paclitaxel-resistant cancer cells [200]. It is observed that FOXO3a in glycolysis increases the expression levels of P-glycoprotein/ABCB1 which drives docetaxel cross-resistance. Knockdown of FOXO3a expression levels impairs glycolytic shift-induced apoptosis. Targeting of FOXO3a in glycolysis restores the sensitivity of the docetaxel-cross-resistant paclitaxel-resistant cancer cell line and triggers apoptosis [200]. Lin and his co-workers [201] demonstrated that there is a partial cross-resistance between paclitaxel and docetaxel. They observed that docetaxel has a modest activity in breast cancer patients (25%) pre-treated with paclitaxel [201].

### Cross-resistance between docetaxel and cabazitaxel

Chen and his co-workers [202] used cabazitaxel to treat docetaxel-resistant prostate cancer. It is observed that cabazitaxel has a potent cytotoxic effect on HCC cell lines by arresting the cancer cell cycle in the G2/M phase and inducing apoptosis in vitro and in vivo. Cabazitaxel also has very low cross-resistant in P-gp-overexpressed HCC cells by 1.53 resistance fold compared to paclitaxel and docetaxel [202]. Lombard and his coworkers [203] declared that the docetaxel resistance induces cross-resistance to cabazitaxel. It is observed that high expression levels of ABCB1 in docetaxel-resistant prostate cancer cells are the main cause of cross-resistance to cabazitaxel [203]. ABCB1 inhibitors involving antiandrogen can resensitize taxane-resistant cells to both docetaxel and cabazitaxel. Liu and his colleagues [204] observed that the kinesin-3 superfamily (*KIF14*) associated with cross-resistance between docetaxel and cabazitaxel by inhibiting microtubule depolymerization. The knockdown of the expression levels of *KIF14* drives decreasing the expression levels of AKT and induces the sensitivity of resistant cells towards taxanes [204]. Wang and his coworkers [205] observed that inhibition of the nuclear receptor ROR drives downregulating the expression levels of MDR1 expression. Furthermore, its inhibition restores the sensitivity of cross-resistant cells towards DTX and CBZ. Machioka and his coworkers [206] observed that inhibition of MDR1 restores the sensitivity of cross-resistant cells to DTX/CBZ. Cross-resistance between taxanes is a significant clinical issue that needs more research studies to understand and deal with it.

## Conclusion

The administration of chemotherapy, specifically paclitaxel and its derivatives, are employed as a therapeutic approach for eradicating malignant neoplastic cells. The compounds demonstrate an anticancer mechanism through its interaction with β-tubulin, resulting in the stabilization of microtubules. This stabilization subsequently leads to the arrest of the cancer cell cycle, specifically in the G2/M phase. Following an extended period of administration of paclitaxel and its derivatives, cancer cells develop a resistance to chemotherapy. This review explores the recently discovered mechanisms of taxanes resistance in cancer cells. Furthermore, it provides a comprehensive list of genes associated with resistance to taxanes. miRNAs list related to taxane resistance and cross-resistance mechanisms between taxanes.

## Data Availability

No datasets were generated or analysed during the current study.
